# Supplementary material for: Polymer gels with tunable ionic Seebeck coefficient for ultra-sensitive printed thermopiles
Source: Nat Commun. 2019 Mar 6;10:1093. doi: 10.1038/s41467-019-08930-7 (PMC6403253; doi:10.1038/s41467-019-08930-7)
Supplement: Supplementary file 1 — Supplementary Information [file 41467_2019_8930_MOESM1_ESM.pdf]

Supplementary Information for

Polymer gels with tunable ionic Seebeck coefficient for ultra-sensitive printed thermopiles

Zhao et al.

Supplementary Figures

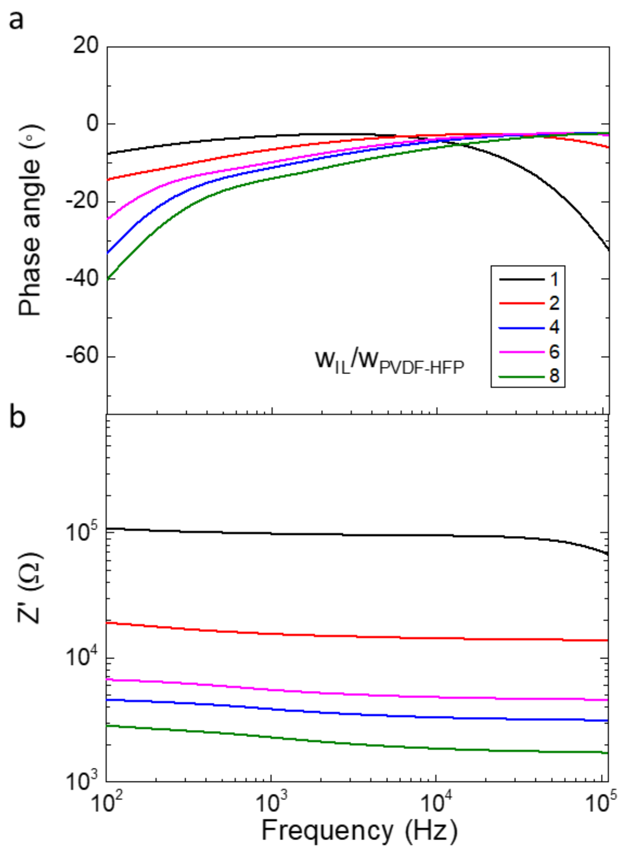

Supplementary Figure 1. Impedance spectroscopy of [EMIM][TFSI]/PVDF-HFP polymer gels. **a** Phase angle and **b** real part of the dielectric function as a function of frequency for the polymer gels with different $w_{IL}/w_{PVDF\text{-}HFP}$ weight ratio.

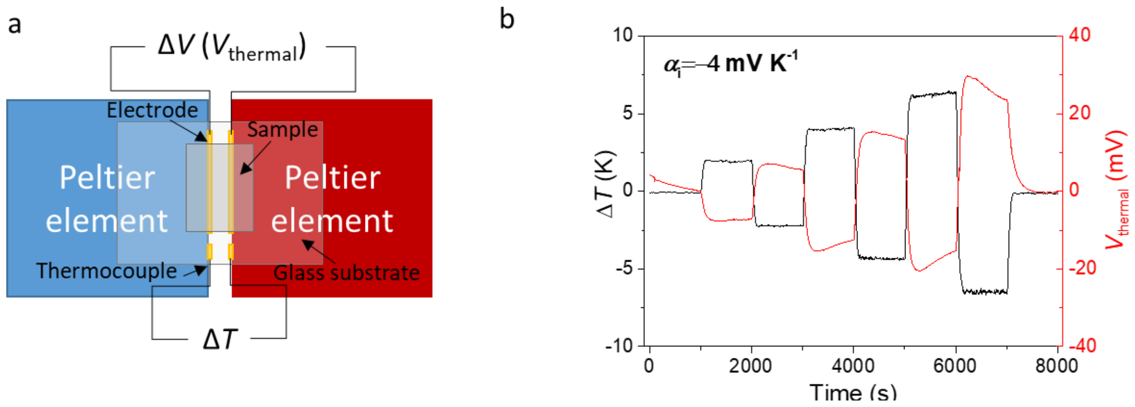

Supplementary Figure 2. Thermoelectric properties of [EMIM][TFSI]/PVDF-HFP polymer gels. **a** Schematic of the Seebeck coefficient measurement setup. **b** Temperature difference (ΔT) and thermovoltage measurement (V_{thermal}) results.

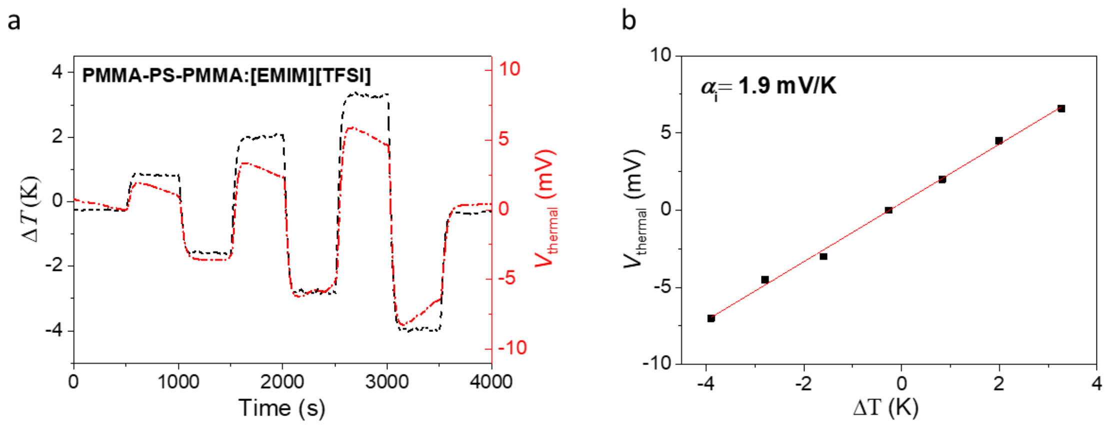

Supplementary Figure 3. Thermoelectric property of [EMIM][TFSI]/PMMA-PS-PMMA polymer gels. **a** V_{thermal} changing with ΔT from -7 K to 6 K of [EMIM][TFSI]/PMMA-PS. **b** Linear fitting of V_{thermal} with ΔT .

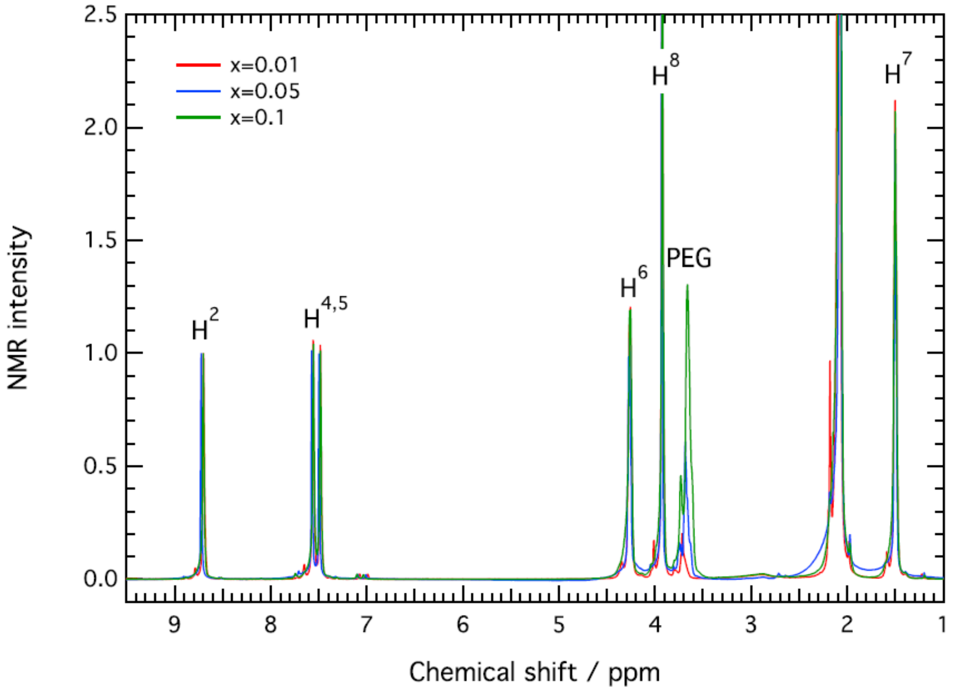

Supplementary Figure 4. ^{1}H NMR spectra of the polymer gel. All ^{1}H NMR spectra revealed, along with the characteristic resonances of [EMIM][TFSI], an additional signal at ~2 ppm, which we assign to traces of acetone (three H nuclei per molecular unit). That a few molecules of acetone are still present in the gel is a consequence of preparing the gels inside NMR tubes, which results in a more difficult evaporation of the solvent than for gels over a glass. The alternative approach of preparing films/gels over glass slides for a post-synthesis transfer into NMR tubes, however, has the more severe drawback of structural inhomogeneity, which can disable the correct estimation of self-diffusion values. The additional peak assigned to acetone has a constant intensity with respect to the IL-characteristic signals. With these insights, we conclude that self-diffusion values are larger than expected due to the fluidizing effect of acetone[1], but that this effect is a constant through the series of gels investigated. In other words, the trend changes that we measure and discuss are a true effect of adding PEG.

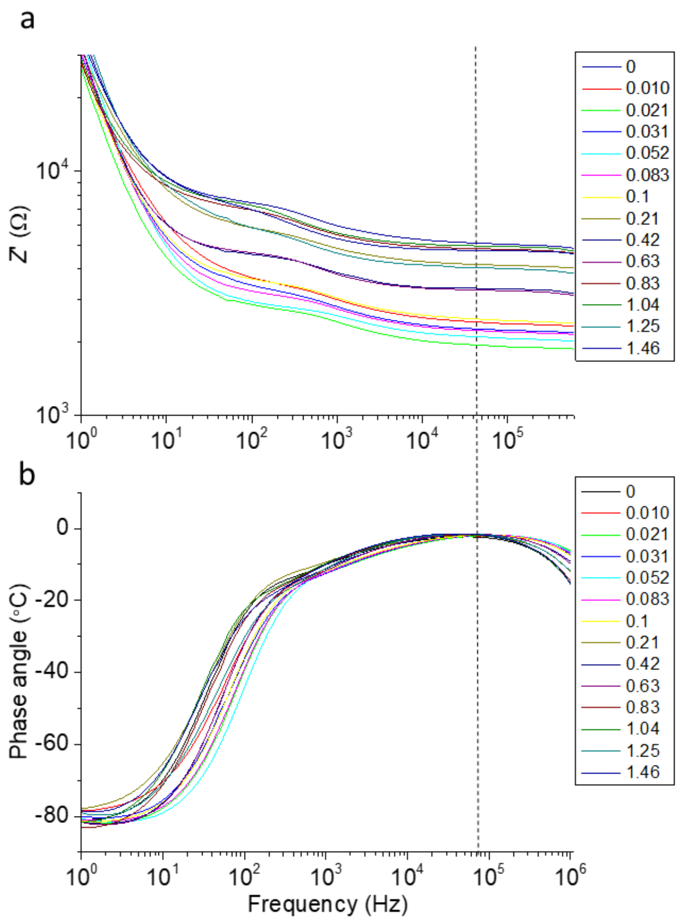

Supplementary Figure 5. Impedance spectroscopy of the IL-p gel with different content of PEG. **a** Real part of the impedance and **b** phase angle as a function of frequency from 1 Hz to 1 MHz.

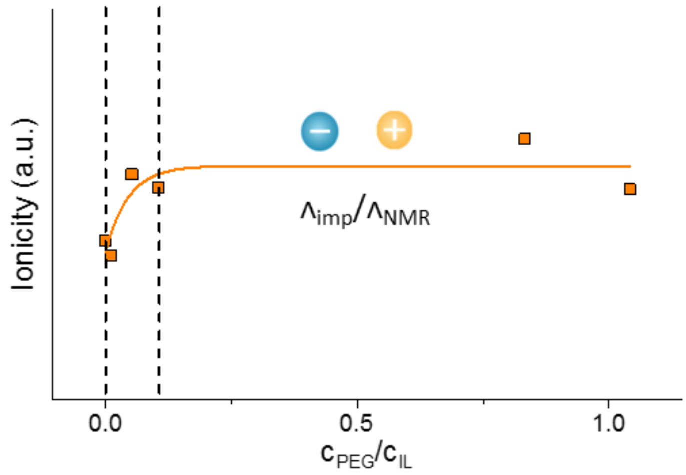

Supplementary Figure 6. Fraction of the dissociated ions (ionicity) of IL-p gels with different PEG content.

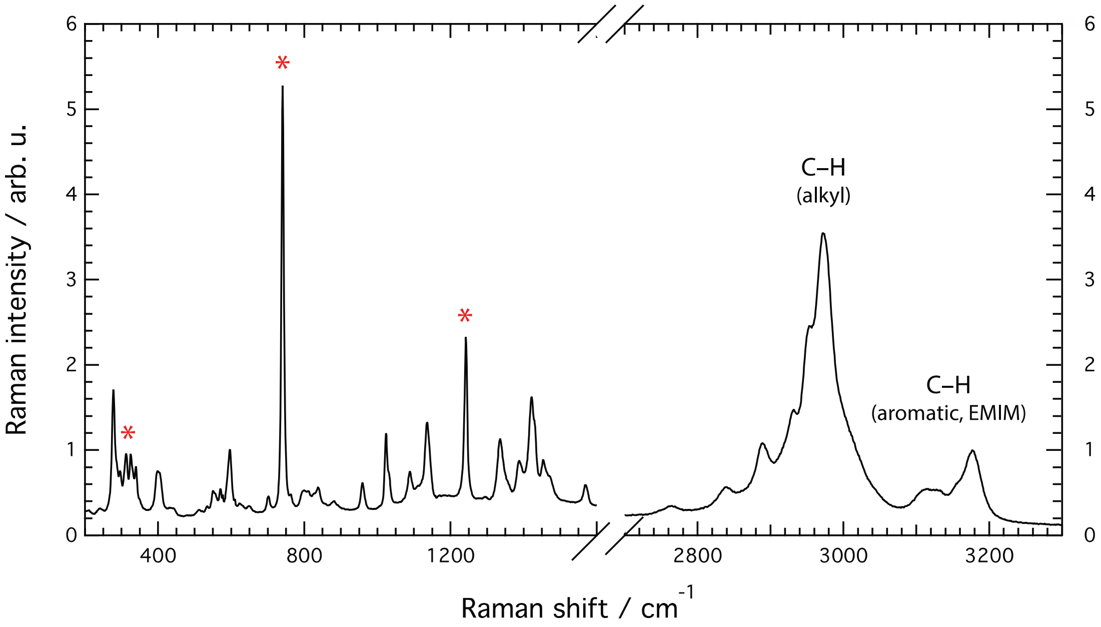

Supplementary Figure 7. Raman spectrum of the polymer gels. The spectrum is recorded at room temperature in the back-scattering geometry, with the incoming laser reaching the gels/films from above. Asterisks mark vibrational modes characteristic of the TFSI anion, while the C-H stretching modes of alkyl chains (ethyl and methyl in EMIM and ethyl in PEG) and of the aromatic ring ($C^{2,4,5}$-H in EMIM) are observed in the frequency ranges 2800-3100 and 3100-3200 cm^{-1} respectively. The Raman spectrum for the gel with c_{PEG}/c_{IL}=0.052 is shown below as a representative case. A detailed peak-fitting procedure was employed to the regions 720-760 cm^{-1} and 3040-3240 cm^{-1} to extract the position of the TFSI-characteristic mode at 740 cm^{-1} and the C^2-H and $C^{4,5}$-H stretching mode of the EMIM cation. The latter are known to red shift as a result of stronger $C^{2,4,5}$-H···O or $C^{2,4,5}$-H···N hydrogen bonds, whereas a red shift of the 740 cm^{-1} mode is normally associated to weaker TFSI···X interactions. These shifts as a function of PEG content are reported in the main text of the paper.

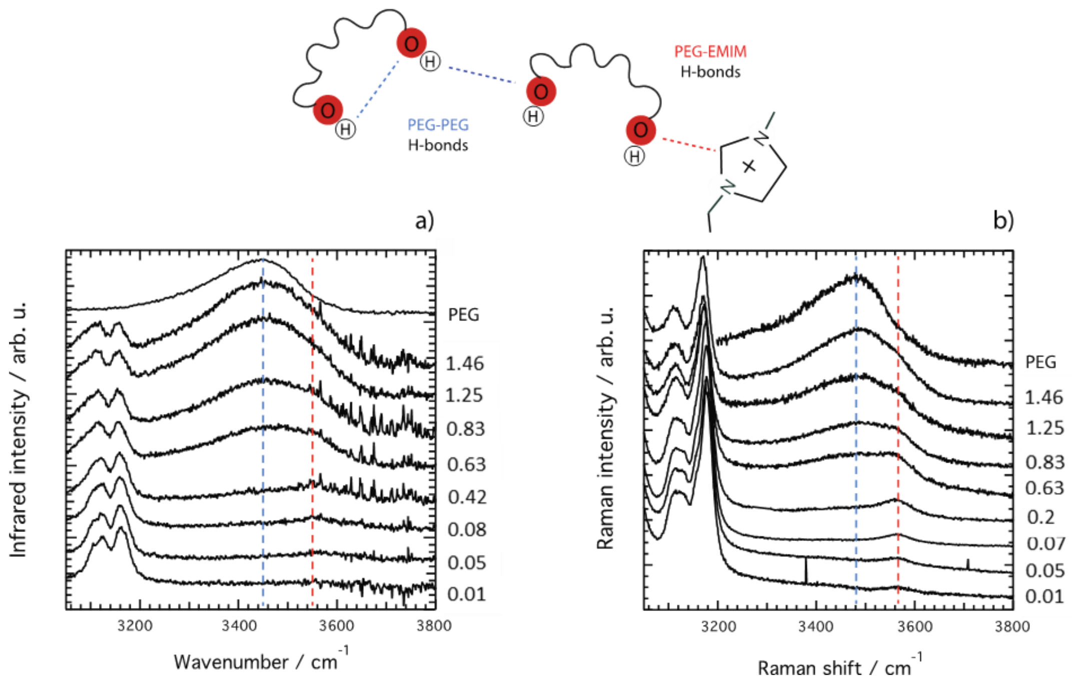

Supplementary Figure 8. Infrared and Raman spectra recorded for the gels at increasing content of PEG showing the spectral range of O-H stretching. Both Raman **a** and infrared **b** spectroscopy show a feature comprising two components, one at higher frequencies (red lines) representative for weakly interacting OH groups, and another at lower frequencies (blue lines) representative of more strongly hydrogen bonded OH groups. The lower frequency component is dominant in pure PEG in which both inter- and intra-molecular hydrogen bonds are formed. In the gels, we observe a gradual increase of the lower frequency component with increasing PEG content.

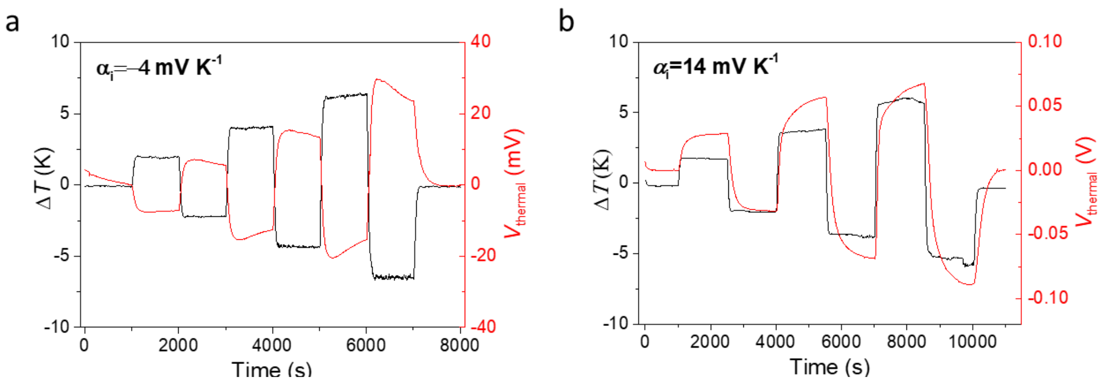

Supplementary Figure 9. Switch of Seebeck coefficient of IL-p gels by directly adding PEG. First the IL-p gel film was prepared as previously mentioned, the Seebeck coefficient was measured as shown in **a**. Then a few drops of PEG were directly placed on the surface of the sample. After 6-12 hours, the extra PEG was removed by clean fabric paper, and the Seebeck coefficient was measured as shown in **b**.

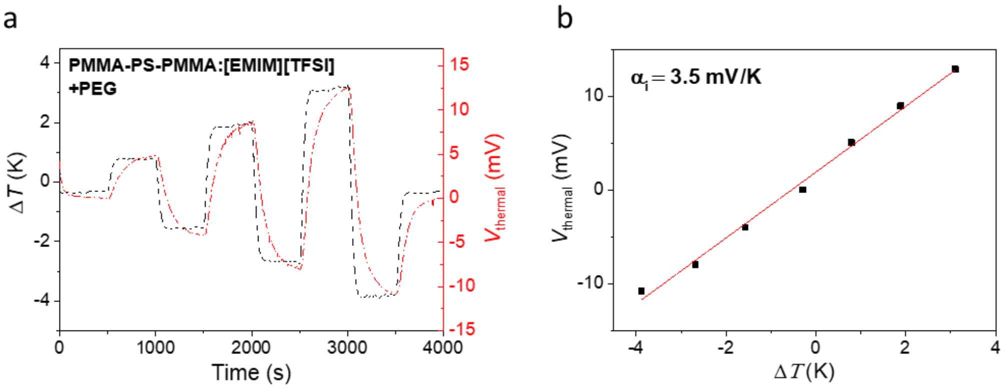

Supplementary Figure 10. Seebeck coefficient of [EMIM][TFSI]/PMMA-PS-PMMA after adding PEG. **a** $V_{thermal}$ changing with ΔT from -7 K to 6 K. **b** The linear fitting of $V_{thermal}$ with ΔT.

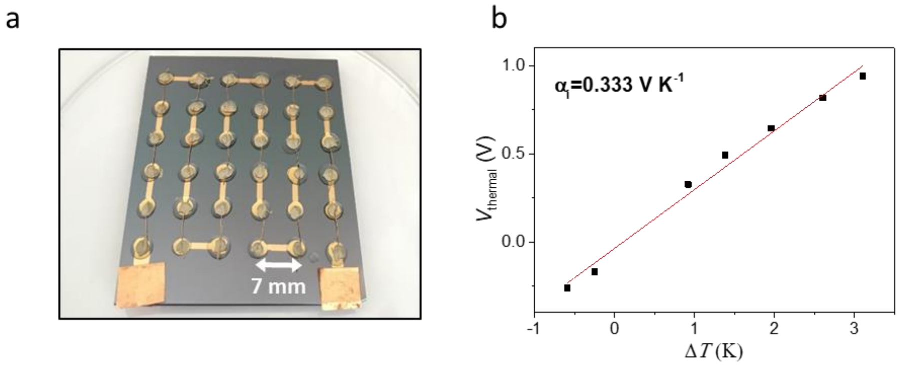

Supplementary Figure 11. Seebeck coefficient of the integrated device with 18 pairs of legs**.** **a** Photograph of the device with 36 legs connected. **b** Linear fitting of V_{thermal} changes with ΔT of the final device (thickness of 80±10 μm).

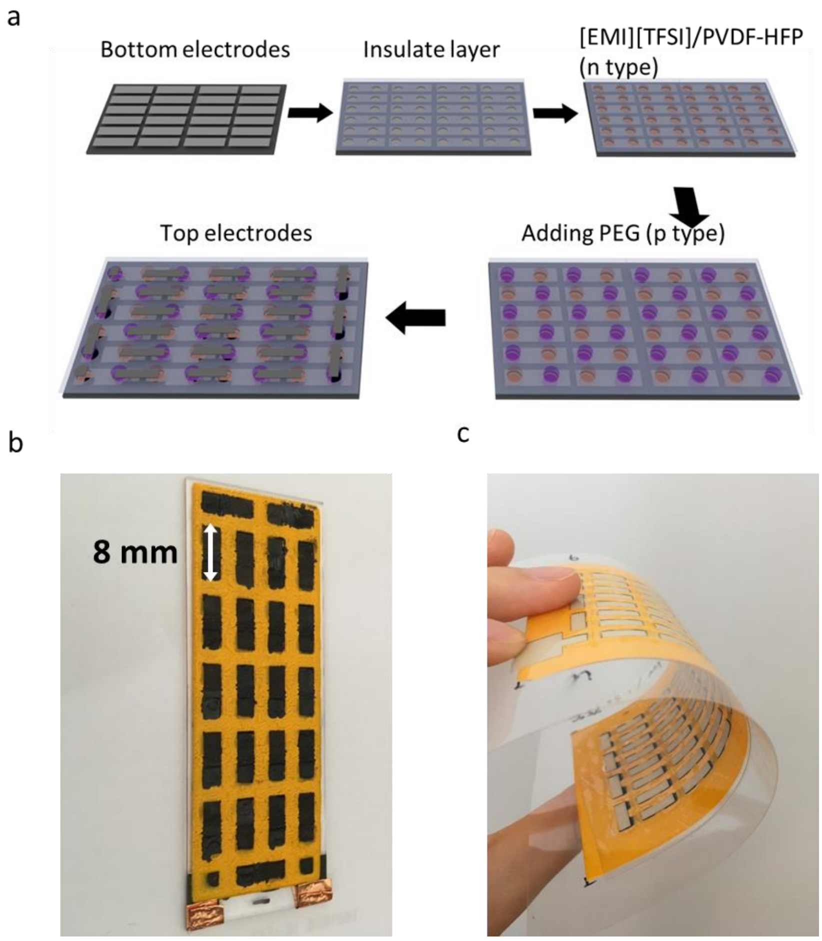

Supplementary Figure 12. Screen printing of the device. **a** Illustration of the screen-printed device. Photographs of the printed devices on **b** glass substrate and **c** plastic substrate.

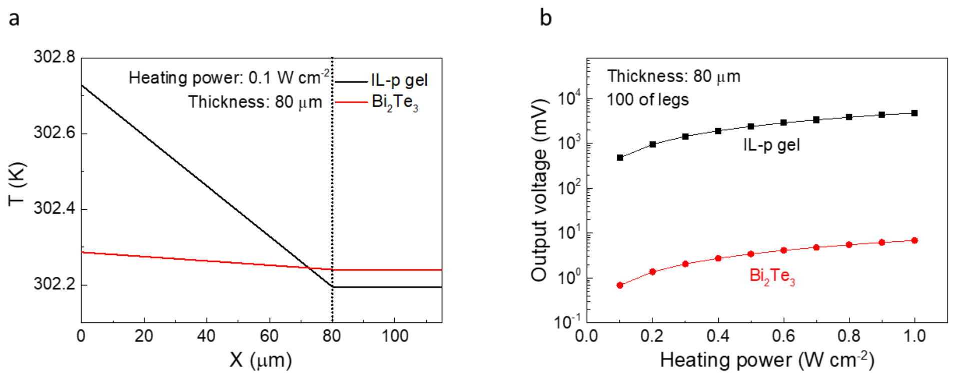

Supplementary Figure 13. Simulations of temperature distribution and calculated output voltage of the IL-p gel and Bi_2Te_3. **a** Simulation of temperature distribution in IL-p and Bi_2Te_3 film with the same structure and the same heating power of 0.1 W/cm^2. **b** Calculated output voltage of thermoelectric modules composed of IL-p gel and Bi_2Te_3 film plotted with different heating power. Finite element method (Comsol Multiphysics 5.0) was used to simulate the temperature distribution across the IL-p gel (80 μm thick) on top of the Si wafer (500 μm thick coated with 1μm SiO_2 on both sides) when heated from the top surface using a 0.1 W/cm^2 heat source. As comparison, we simulated the same system, but with the gel replaced with the same thickness of Bi_2Te_3. Material properties for the IL-p gel were as follows: density=1.89 g/cm^3, thermal conductivity=0.136 $W/(m \cdot K)$ and heat capacity=1.27 J/K/g (measured by differential scanning calorimetry[2] at room temperature). For the other materials, we used material properties from the built-in material database of the software. A one-dimensional system was used to resemble an infinitely wide system. Convective heat flux was employed at the top and bottom boundaries of the sample, with heat transfer coefficients of 10 W/m^2 K at the top of the thermoelectric layer (to resemble air environment) and 100 W/m^2 K at the bottom of the wafer (to approximate heat transfer to the heat sinking structure). Surrounding temperature was set to 293.15 K.

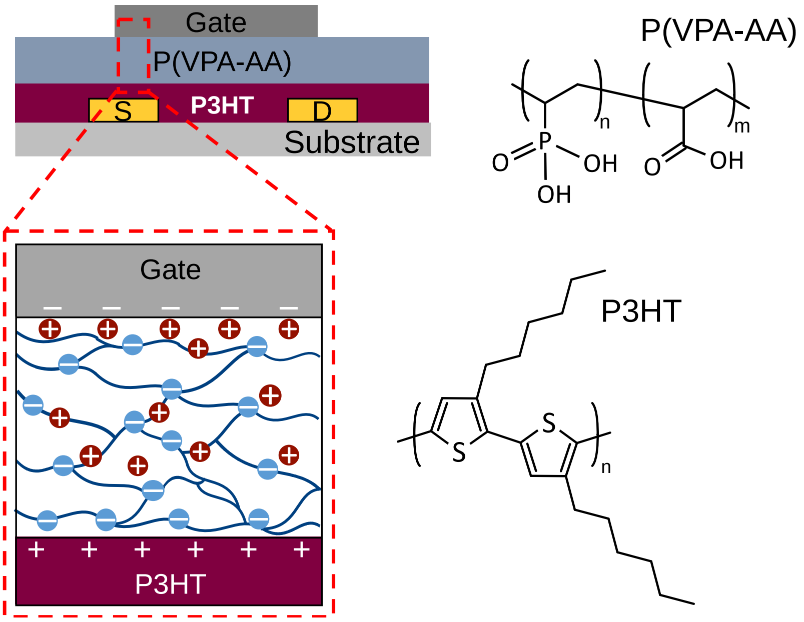

Supplementary Figure 14. Structure of the electrolyte gated transistor. Schematic diagram of an electrolyte-gated transistor and illustration of the channel charge and ion distribution within the electrolyte layer. The chemical structures of P3HT and P(VPA-AA) are also shown. Top gate, bottom contact transistors based on regioregular poly(3-hexylthiophene-2,5-diyl) (P3HT) are fabricated using poly(vinylphosphonic acid-*co*-acrylic acid) (P(VPA-AA)) as the polyanionic electrolyte insulator. The P(VPA-AA) phosphonic acid groups provide plenty of potentially mobile cations. The anionic phosphonate groups are instead virtually immobile, preventing penetration of anions into the conjugated polymer when a negative gate is applied. When a negative bias voltage is applied to the gate, protons in the electrolyte insulator layer drift the electrolyte/semiconductor interface to the electrolyte-metal gate interface, and establish two electrical double layers (EDLs) across which the entire gate potential drops.

Supplementary Notes

Supplementary Note 1. Calculation of the amount of charge in the polymer gel.

The Seebeck coefficient does not depend on the total mobile ions, but it relates to the temperature dependence of the structural entropy, which is related to the interactions between ions and solvent along the thermal field. In order to show that it is possible for the ionic liquid gels in the studied concentration range to have similar Seebeck coefficient, we have calculated the total number of ions in the lowest IL content sample, and the number of ions needed to reach thermal voltage of 40 mV (consider ΔT of 10K is applied to the two electrodes). The thickness of the gel film is 80 μm, the length and distance between two electrodes are 200 mm and 1 mm respectively, and the molar concentration of the sample with IL/PVDF-HFP = 0.8 is 1.88 mmol cm^{-3}, so the number of ions is around 2.2×10^{19}. For pure IL liquid, the molar concentration is 3.88 mmol cm^{-3}, only more than 2 times than the lowest ratio (0.8) in our study range. To reach thermal voltage of 40 mV between the two electrodes, Q=CV is needed, where C is the capacitance of Au (typically 1μF cm^{-2}). It turned out that only 4.9×10^{6} ions are required to obtain the open voltage, which is far lower than the total number of ions in the samples. Hence, although the total amount of ions can greatly affect the ionic conductivity, the ionic Seebeck coefficient can stay within a narrow range.

Supplementary Note 2. Thermal conductivity of the IL-p gel

The thermal conductivity of the IL-p gel is estimated using the effective medium theory[3] for composite. Based on the known thermal conductivity of [EMIM][TFSI] (0.12 W/m·K) and PVDF-HFP (0.2 W/m·K) at room temperature, we can calculate the thermal conductivity of IL-p gel through the formula $\kappa_c=\kappa_p\phi_p+\kappa_w\phi_w$ (κ and ϕ are thermal conductivity and weight ratio, the subscripts c, p and w correspond to the gel, ionic liquid and PVDF-HFP respectively). The calculated thermal conductivity of the IL-p gel is 0.136 W/m·K.

Supplementary References

[1] Rausch, M. H., Hopf, L., Heller, A., Leipertz, A., Fröba, A. P., Binary Diffusion Coefficients for Mixtures of Ionic Liquids [EMIM][N(CN)2], [EMIM][NTf2], and [HMIM][NTf2] with Acetone and Ethanol by Dynamic Light Scattering (DLS), J. Phys. Chem. B, 117, 2429-2437 ,(2013); RSC Adv., Kalugin, O. N., Riabchunova, A. V., Voroshylova, I. V., Chaban, V. V., Marekha, B. A., Koverga, V. A. Idrissi, A., RSC Adv., 206, 6, 8906-8912, (2013).

[2] Wang, Y. Q., Chung, M. T., Oneal, N. J., Brill, W. Differential scanning calorimetry at charge-density-wave transitions, Synthetic Metals 1992, 46, 307.

[3] Ail, U., Jafari, M. J., Wang, H., Ederth, T., Berggren, M., Crispin, X., Thermoelectric Properties of Polymeric Mixed Conductors, Adv Funct Mater, 26, 6288-6296, (2016).
